# Factors associated with access to health care among foreign residents living in Aichi Prefecture, Japan: secondary data analysis

**DOI:** 10.1186/s12939-021-01465-8

**Published:** 2021-06-10

**Authors:** Michiyo Higuchi, Maki Endo, Asako Yoshino

**Affiliations:** 1 Department of Global and Community Health, Nagoya City University School of Nursing, 1 Kawasumi, Mizuho-cho, Mizuho-ku, Nagoya, 467-8601 Japan; 2 Nagoya City University Hospital, 1 Kawasumi, Mizuho-cho, Mizuho-ku, Nagoya, 467-8601 Japan

**Keywords:** Migrants, Access to health care, Universal health coverage, Japan, Non-governmental organization

## Abstract

**Background:**

In Japan, foreign residents, and particularly new arrivals in the country, experience barriers to health care and show poorer health outcomes when compared to Japanese nationals. The health-care-related situation for foreign residents in Japan has been characterized by drastic changes over time; thus, there is difficulty identifying individuals who are “left behind” by the system. In this study, we aimed to identify, among foreign residents who attended informal free medical consultations, factors associated with “being advised to visit a medical facility” and “being referred to a medical facility,” which represented hypothetical proxy indicators of barriers to health care.

**Methods:**

Secondary data analyses were conducted using the activity records of a non-governmental organization that provides free consultations targeting foreign residents in various locations in Aichi Prefecture, Japan. Participant characteristics, including insurance coverage, were determined. Bivariate and multi-variate analyses were performed to identify factors associated with having barriers to health care.

**Results:**

Among 608 extracted cases, 164 (27.5%) cases were advised to visit a medical facility, and 72 (11.8%) were referred to a medical facility during the consultations. Those who were not covered by public insurance showed a 1.56-time (95% confidence interval [CI]: 1.19–2.05) higher prevalence of being advised to visit a medical facility when compared to those who were covered by public insurance. Unemployed people and students were more likely to be referred to a medical facility than were professional workers; the prevalence ratios were 3.28 (95% CI: 1.64–6.57) and 2.77 (95% CI: 1.18–6.46), respectively.

**Conclusions:**

Although the majority were insured, almost 30% were advised to visit a medical facility, which implied that they had had limited access to the formal health-care system before availing of the free consultations. The findings highlight those uninsured, unemployed people and students, who are considered vulnerable to access to health care. It is vital to provide those who are vulnerable with the necessary support while updatinge evidence, so that no one is “left behind.”

## 他言語要約 (日本語)

**背景:**

日本において、外国人住民は日本人に比べて保健医療へのアクセスバリアがあり、健康のアウトカムもよくないことが知られている。しかし、外国人住民の保健医療に関する状況は大きく変化し続けており、「取り残された」人を特定することは困難である。そこで本研究では、NGOの無料健康相談を受けた外国人住民を対象に、「医療機関を受診するように勧められた」「医療機関を紹介された」ことを保健医療へのアクセスバリアを示唆する代理指標として、これらに関連する要因を明らかにすることを目的とした。.

**方法:**

愛知県内の複数の会場で外国人住民を対象とした無料健康相談会を行っているNGOの活動記録を用いて二次データ分析を行った。保険加入を含む参加者特性を明らかにした上で、二変量分析(χ^2^検定) および多変量分析 (log-binomial回帰分析)を行い保健医療へのアクセスバリアと関連する要因を明らかにした。.

**結果:**

抽出された608例のうち、相談時に医療機関への受診を勧められたのは164例(27.5%)、医療機関への紹介は72例(11.8%)であった。公的保険に加入していない人は、公的保険に加入している人に比べて、医療機関への受診を勧められる割合 (prevalence)が1.56倍(95%信頼区間 [CI]:1.19–2.05)高かった。仕事をしていない人および学生は、専門職者に比べて医療施設への紹介を有意に多く受けており、prevalence ratioはそれぞれ3.28(95%信頼区間 [CI]:1.64–6.57) および2.77(95%CI,1.18–6.46) であった。.

**結論:**

大多数は保険に加入していたが、無料健康相談を利用する前に正式な医療制度へのアクセスが制限されていたことを示唆する、医療機関を受診するよう勧められた参加者が30%近くいた。本研究結果は、保険に加入していない人、仕事をしていない人、学生など、保健医療へのアクセスが脆弱だと考えられる人たちを示した。誰もが「取り残されない」ようにするために、エビデンスを更新しながら、脆弱な人々へ必要な支援を提供することが肝要である。.

## Background

“Leave no one behind,” which represents the goal of eradicating poverty, discrimination, and exclusion and of reducing inequalities and vulnerabilities, is a core element of the global community’s agenda [1]. To help realize this vision, universal health coverage (UHC) has been included as a target of the United Nation’s Sustainable Development Goals, and achieving UHC has become a priority in the health sectors of many countries [2]. In this global context, Japan is recognized as a country where universal public insurance coverage was implemented in the country almost 60 years ago [3]. However, despite this long-term existence of the universal health insurance systemin Japan, a health gap exists, with some population groups having little access to coverage [4]. In particular, migrants and immigrants are considered to be vulnerable groups in regard to both access to health care and overall health [5–8].

Up to March 2019, Japan maintained a strict policy regarding foreign workers, except those who held visas based on their civil status (this is officially termed “based on status or position”) and or those engaged in professional work or designated activities. Approximately 40 years ago, few foreign residents lived in Japan with the exception of “special permanent residents”; these were individuals (and their descendants) who stayed in Japan after losing their Japanese nationality when the Treaty of San Francisco took effect. Since the 1980s, however, the number of “newcomers” has been increasing, mainly as a result of various socio-economic circumstances, such as a shortage of labor in Japan and the country’s buoyant economy [9, 10]. In 1990, the Japanese government adopted a new policy regarding immigrants with Japanese heritage, enabling them and their families to obtain residence status [11–13]. In 1993, a new residential qualification, “technical intern,” was established. Technical interns are not officially laborers; however, they comprise part of the workforce in Japan [12, 14]. Another surge of newcomers has been the “students” who came to Japan as a result of the government’s “300,000 International Students Plan,” which was introduced in 2008 [15, 16]. In 1990, the total number of foreign residents in Japan exceeded one million, and 15 years later it reached two million [17]. By the end of 2019, foreign residents numbered 2,933,137 [18], representing 2.3% of the total population in Japan. “Newcomers” have outnumbered special permanent residents since the early 1990s [19]. Among all foreign residents, “general permanent residents” (permanent residents other than special permanent residents) are now the most prevalent group (27.0%), followed by “technical interns” (14.0%), and “students” (11.8%) [18].

According to the latest “basic survey on wage structure” by the Ministry of Health, Labor and Welfare, Japan [20], the average monthly wage among foreign laborers was 223 thousand JPY whereas the total average was 308 thousand JPY. The duration of employment of the survey populations was different from each other (3.1 years whereas 12.4 years, respectively); however, compared with the average wage among the whole laborers in the age stratum of 30–35 years old, the average age of foreign laborers (33.4 years old in average) was still lower. Also, foreign laborers’ wages varied much among resident status. Workers with status/position-based visa (permanent residents, long-term residents and spouses or children of Japanese nationals or permanent residents) earned lower than professional and technical workers. The technical interns’ average wage was less than a half of one of the professional and technical workers.

Japanese society must now respond to this rapid increase in the total number as well as diversity of foreign residents to ensure that people can coexist in the country [21]. In this regard, maintaining the their health is one of the most urgent social issues. Therefore, it is needed to understand access to health care among foreign residents and its associated factors. A study on access to health care among Nepalese migrants in 10 prefectures of Japan used two indicators; perceived better access to a doctor/health worker and needed to see a doctor/health worker, but did not. No need of Japanese interpreter during visit to health facilities and payment of the health insurance premium regularly were positively associated with both indicators. The length of stay in Japan and self-rated health were also positively associated with perceived better access [8]. Our team’s previous study applied “no regular doctor” and “unmet health care needs” as negative access indicators and surveyed Filipino women in Aichi prefecture. In the study, emotional/informational social support was associated with both indicators. Tangible social support, having a Japanese husband, and Japanese language proficiency were also associated with no regular doctor [22]. Some previous studies investigated factors associated with access to health-related resourses among specific groups but not directly focused on access to health care; for example, access to HIV testing among international students in Japanese language schools in Tokyo and access to health insurance among Latin American immigrants in a city [7, 23]. In Japan, there are few studies on access to health care among foreign residents. To our best knowledge, there is no quality study on access to health care among foreign residents in general in Japan.

In addition to the formal arrangements made by local governments, non-governmental organizations (NGOs) and non-profit organizations (NPOs; hereafter simply referred to as “NGOs”) provide support for foreigners’ health. Some of these NGOs provide informal, free medical check-ups and consultations. These NGO-organized free check-ups/consultations (hereafter referred to as “free consultations”) are external to the formal health system in Japan [24, 25]. The free consultations are not usually held in medical facilities (instead, public buildings such as community centers, schools, and churches are used); this means that the volunteer doctors who participate in these consultations do not have access to the appropriate equipment to provide full services for attendees. These NGOs visit various locations in their areas, typically visiting each place once or twice a year, and offer simple examinations and advice. During the free consultations, some attendees are advised to visit a medical facility that is part of the formal health system in order to undergo further examination and/or receive treatment. As people with no barriers to obtaining health care through the formal health system would be likely to visit official medical facilities rather than wait for a free consultation, it can be assumed that attending free consultations and being referred to a medical facility indicates that the attendee faced a barrier to visit a medical facility until they had the opportunity to attend the free consultation.

In the present study, using a dataset from an NGO that provides free medical check-ups in Aichi Prefecture (which has the second-largest foreign population in Japan [18], we aimed to identify, among foreign residents who attended free consultations, factors associated with “being advised to visit a medical facility” and “being referred to a medical facility”; these were considered to represent negative indicators of access to health care. Major characteristics of the Japanese health care system are universal health insurance and free access [26]. Anyone who stays in Japan for over three months should be covered by a public health insurance scheme [27] and can access any medical facility in the formal health care system [26]. Therefore, it is reasonable to assume that there is a barrier to health care in daily life if somebody, regardless of Japanese nationals or not, consults informal health services and holds a health problem which requires to visit the formal health care system at the point of an informal consultation. Replies from referral facilities were also reviewed to determine whether participants, after their referral, had entered the formal health care system. As studies on access to health-care among foreign residents in Japan remain limited, the current study will provide knowledge regarding the individuals who are actually “left behind,” as well as information about the support such people require.

## Methods

### Study design

Secondary data analyses were conducted using the activity records of an NGO located in Aichi Prefecture, Japan.

### Data source

The NGO, Medical Information Center, Aichi (MICA), has provided foreign residents with medical information and free monthly consultations since 1998. Supported by volunteer doctors, dentists, and nurses, as well as non-medical volunteers, the free consultations are provided in various locations in Aichi Prefecture, with each location being visited in rotation. The documentation for these consultation activities comprises a four-page form that the MICA staff members and volunteers complete for each attendee. The pages cover: 1) personal information, including sex, age, date of birth, nationality, occupation, years living in Japan, whether the attendee is covered under a Japanese public insurance scheme, medical history, family medical history, complaints and symptoms; 2) lifestyle, 3) data obtained through physical check-ups (height, weight, blood pressure, pulse rate, body temperature, and urine analysis results) and the results of the consultation with the doctor, including an overall assessment (i.e., the actions the attendee should take); and 4) oral check-ups and the results of consultations with a dentist, with an overall assessment (the actions the attendee should take). The form is available in 10 different languages (English, Portuguese, Spanish, Chinese, Korean, Vietnamese, Filipino, Indonesian, Burmese, and Thai), which ensures that most attendees can understand the questions. All of the information gathered on these forms is entered into a data-management program by MCIA staff members.

### Participants

For the present study, anonymous data of all adults (aged 18 years or older) who participated in the free monthly consultation activities between 2012 and 2016 were extracted from the full dataset; this was performed under the terms of a data-use agreement between the first author and the NGO (MICA). We used 2012 as the start date for the data extraction because in 2012 the Japanese government made a legislative change that allowed foreign residents who lived in Japan for over three months to apply for National Health Insurance (previously, only foreign residents who had lived in Japan for over one year were eligible.) Cases were excluded if the age was unknown (four cases), nationality was Japanese (16 cases), the participant was a traveler (three cases), and if all records except attributes were missing (one case). Thus, of a total of 632 cases, 608 were used for the analyses.

### Access to health care framework

There are various models and frameworks to consider access to health care [28–30]. For example, the model published by the Institute of Medicine in the United States [31] applies “use” of services as the intermediate indicators of its access to health care model. Andersen and colleagues [32, 33] have developed several frameworks over the years. In their frameworks, both individual characteristics and contextual characteristics influence health care access. Penchansky and Thomas [34] defined access as entry into or use of the health care systems and suggested specific dimensions, such as availability, accessibility, accommodation, affordability and acceptability, to describe the “fit” between the patient and health care system. Levesque et al. [30] suggested access as “opportunity” based on the review of previous studies. International surveys often use a question asking “unmet health care needs” [35], which are also often used in studies on nationals and migrants in Japan [22, 23, 36].

### Variables measurement

#### Explanatory variables

All available participant characteristics included in the dataset, such as sex, age group, region of origin, occupation, whether he/she was covered by a Japanese public insurance scheme, and time living in Japan, were used as explanatory variables. Supply-side variables were not included in the dataset we used. Therefore, only attendee (demand-side) variables were included as explanatory variables in our analysis model.

Region of origin was defined based on the nationality recorded on the form and categorized based on United-Nations-recognized regions, unless the nationality was Brazilian, Chinese, Filipino, Vietnamese, or a high-income country that is defined by the World Bank. As Brazilian, Chinese, Filipino, and Vietnamese were the four major groups, they were treated as a single category. “Other Asia” featured 68 participants from South Asia (five countries), 34 from South-East Asia, except Vietnam and the Philippines, (three countries), two from West Asia (one country), and one from Central Asia (one country). Participants included in the “other” region were as follows: 35 from Latin America, except Brazil, (five countries), six from East Africa, three from West Africa, three from North Africa, one from Central Africa, two from Russia, and one from Melanesia.

In the data-collection form,  attendee's occupation was determined using an open-ended, self-reported answer and, for the present study, we developed occupational categories based on these answers. If the type of factory work or construction work was recorded, or if the occupation was simply recorded as “factory,” the participant was categorized as engaging in “factory or construction work.” If part-time or contingent work (*Arubaito*, *Paato*, *or Haken*, in Japanese) was recorded as an occupation, the participant was categorized as having a “non-regular job.” “Trainee” and “intern” were combined to form “trainee/intern.” These three categories were then further grouped into the category “labor and non-regular,” because they often overlap and are used interchangeably in daily conversation. Professional workers, such as teachers, engineers, and company workers, were also combined because they are considered to represent the formal sector. Housewives and the unemployed were combined because these people usually stay home and, again, these terms are sometimes used interchangeably in daily conversation. “Other” included nine people engaged in “service,” seven helpers (care assistants), four people engaged in religion-related work, three salespeople, three businesspeople, three cooks, two refugees, two volunteers, one babysitter, and one diplomat. Two occupation records could not be categorized, and were also included in “other.”

#### Outcome variables

All cases for which there were any records on the third page of the form were defined as “had a medical consultation.” Two items were used as the outcome variables and considered as indicators of having barriers to health care: 1) “being advised to visit a medical facility” (attributed if the participant was advised to visit a medical facility); and 2) “being referred to a medical facility” (attributed if the participant was referred to a medical facility, with or without a referral letter). We assumed that participants who were advised or referred did not have easy access to the formal health-care system before attending the NGO’s consultations. Participants identified as 2) were part of 1), but were considered more serious cases.

### Data analysis

First, participant characteristics (sex, age group, region of origin, occupation, whether he/she was covered by a Japanese public insurance scheme, and time living in Japan) were described. The frequency and the percentage of each category were calculated. Second, the two indicators of barriers to health care (“being advised to visit a medical facility” and “being referred to a medical facility”) were analyzed in terms of participant characteristics using chi-square tests. Third, log-binomial regression analyses were performed to calculate the prevalence ratios (PRs) of “being advised to visit a medical facility” and “being referred to a medical facility” for each participant characteristic. Variables that showed, in the chi-square tests, statistical associations for each of the two access indicators were included in the model. Furthermore, if the participants were referred to a medical facility with or without a referral letter, the recorded consequences were described. Stata SE version 12.1 (Stata Corp) was used for all statistical analyses.

## Results

### Participant characteristics

One-third (34.0%) of the participants were under 30 years of age. Two-thirds of the cases (67.8%) were Vietnamese, Filipino, Brazilian, or Chinese. Almost 30% did not report their jobs. The second-largest occupation group was laborers and non-regular workers (25.0%), followed by professional/company workers (24.3%; the majority of whom were teachers, engineers, and translators). Over one-third (34.7%) had lived in Japan for five years or longer, and almost one-quarter (24.5%) for  ten years or longer (Table 1).

**Table 1:** Participants characteristics among foreign residents who participated in free consultation activities held in Aichi Prefecture from 2012 to 2016 (*N* = 608)

Characteristics	Frequency	%
Sex		
Female	346	56.9
Male	262	43.1
Age group		
-29 years old	207	34.1
30–39 years old	176	29.0
40–49 years old	131	21.6
50- years old	94	15.5
Region of origin		
Vietnam	123	20.2
Philippines	114	18.8
Brazil	105	17.3
China	70	11.5
Other Asia	105	17.3
High-income country	40	6.6
Other	51	8.4
Occupation		
Labour/non-regular^a^	152	25.0
Professional/company	148	14.3
Student	31	5.1
Unemployed^b^	58	9.5
Other	37	6.1
No answer	182	29.9
Duration of living in Japan (years)		
< 1 year	83	13.7
≧1 year, < 5 years	222	36.5
≧5 year	211	34.7
Unknown	92	15.1
Coverage of a Japanese public health insurance		
Covered	497	81.7
Not covered	101	16.6
Unknown	10	1.6

^a^Including trainees/interns

^b^Including housewives

### Associations between access to health care and participant characteristics

Of the 596 participants who had received medical consultations, 164 (27.5%) were advised to visit a medical facility. Occupation category and insurance coverage were associated with being advised to visit a medical facility (*p* = 0.01 and *p* < 0.01, respectively; Table 2). Meanwhile, 72 (11.8%) cases were referred to a medical facility, and the occupation category also showed a statistical association with referral (*p* < 0.01; Table 3).

**Table 2:** Whether advised to visit a medical facility according to participant characteristics among foreign residents who participated in free consultation activities held in Aichi Prefecture from 2012 to 2016 (*N* = 596^a^)

Characteristics	Whether advised to visit a medical facility				*p*-value^b^
	Yes (*N* = 164)		No (*N* = 432)		
	n	%	n	%	
Sex					
Female	100	29.7	237	70.3	0.18
Male	64	24.7	195	75.3	
Age group					
-29 years old	54	26.3	151	73.7	0.36
30–39 years old	41	23.8	131	76.2	
40–49 years old	39	30.5	89	69.5	
50- years old	30	33.0	61	67.0	
Region of origin					
Vietnam	30	24.6	92	75.4	0.59
Philippines	37	33.3	74	66.7	
Brazil	27	26.2	76	73.8	
China	15	22.4	52	77.6	
Other Asia	27	26.0	77	74.0	
High-income country	14	35.0	26	65.0	
Other	14	28.6	35	71.4	
Occupation					
Labour/non-regular^c^	36	24.2	113	75.8	0.01
Professional/company	33	22.5	114	77.6	
Student	9	29.0	22	71.0	
Unemployed^d^	26	46.4	30	53.6	
Other	14	37.8	23	62.2	
No answer	46	26.1	130	73.9	
Coverage under Japanese public insurance^e^					
Covered	121	24.9	366	75.2	< 0.01
Not covered	41	41.0	59	59.0	
Duration of living in Japan^f^					
< 1 year	23	27.7	60	72.3	0.90
≧1 year, < 5 years	59	26.7	162	73.3	
≧5 years	59	28.6	147	71.4	

^a^Twelve participants were excluded because it was not known if they had been advised to visit a medical facility

^b^Chi-square test

^c^Including trainees/interns

^d^Including housewives

^e^Unknown for 9 participants

^f^Unknown for 86 participants

**Table 3:** Whether referred to a medical facility according to participant characteristics among foreign residents who participated in free consultation activities held in Aichi Prefecture from 2012 to 2016 (*N* = 596^a^)

Characteristics	Whether referred to a medical facility				*p*-value^b^
	Yes (*N* = 72)		No (*N* = 524)		
	n	%	n	%	
Sex					
Female	45	13.4	292	86.6	0.28
Male	27	10.4	232	89.6	
Age group					
-29 years old	25	12.2	180	87.8	0.98
30–39 years old	22	12.8	150	87.2	
40–49 years old	15	11.7	113	88.3	
50- years old	10	11.0	81	89.0	
Region of origin					
Vietnam	17	13.9	105	86.1	0.25
Philippines	14	12.6	97	87.4	
Brazil	7	6.8	96	93.2	
China	6	9.0	61	91.0	
Other Asia	19	18.3	85	81.7	
High-income country	4	10.0	36	90.0	
Other	5	10.2	44	89.8	
Occupation					
Labour/non-regular^c^	15	10.1	134	89.9	< 0.01
Professional/company	12	8.2	135	91.8	
Student	7	22.6	24	77.4	
Unemployed^d^	15	26.8	41	73.2	
Other	4	10.8	33	89.2	
No answer	19	10.8	157	89.2	
Coverage under Japanese public insurance^e^					
Covered	57	11.7	430	88.3	0.52
Not covered	14	14.0	86	86.0	
Duration of living in Japan^f^					
< 1 year	10	12.0	73	88.0	0.75
≧1 year, < 5 years	30	13.6	191	86.4	
≧5 years	23	11.2	183	88.8	

^a^Twelve participants were excluded because it was not known if they had been referred to a medical facility

^b^Chi-square test

^c^Including trainees/interns

^d^Including housewives

^e^Unknown for 9 participants

^f^Unknown for 86 participants

### Multiple regression analysis of access to health care

Log-binomial regression analyses suggested that unemployed people, including housewives, showed a higher PR of being advised to visit a medical facility than professional/company workers; the adjusted PR was 2.08 (95% confidence interval [CI]: 1.40–3.11). Among those who were not covered by public insurance, the prevalence of being advised to visit a medical facility was 1.56 times as high as that for participants who were covered by public insurance (95% CI: 1.18–2.05). Unemployed people and students, were more likely to be referred to a medical facility than were professional/company workers; the PRs were 2.77 (95% CI: 1.64–6.57) and 3.28 (95% CI: 1.18–6.46), respectively (Table 4).

**Table 4:** Multiple regression analysis of “being advised to visit a medical facility” and “being referred to a medical facility” among foreign residents who participated in free consultation activities held in Aichi Prefecture from 2012 to 2016

	Being advised to visit a medical facility (*N* = 587^a^)		*p*-value
	aPR^b^	95% CI	
Occupation			
Professional/company	reference		
Labour/non-regular^c^	1.06	0.70–1.60	0.79
Student	1.28	0.69–2.37	0.44
Unemployed^d^	2.08	1.40–3.11	< 0.01
Other	1.47	0.87–2.49	0.15
No answer	1.17	0.79–1.72	0.43
Coverage under Japanese public insurance			
Covered	reference		
Not covered	1.56	1.19–2.05	< 0.01
	Being referred to a medical facility (*N* = 596^e^)		*p*-value
	PR	95% CI	
Occupation			
Professional/company	ref		
Labour/non-regular^c^	1.23	0.60–2.54	0.57
Student	2.77	1.18–6.46	0.02
Unemployed^d^	3.28	1.64–6.57	< 0.01
Other	1.32	0.45–3.87	0.61
No answer	1.32	0.66–2.63	0.43

*PR* prevalence ratio, *aPR* adjusted prevalence ratio

*95% CI* 95% confident interval

^a^Twenty-one participants were excluded because it was not known if a) they were covered by Japanese public insurance or b) if they had been advised to visit a medical facilitys

^b^Mutually adjusted

^c^Including trainees/interns

^d^Including housewives

^e^Twelve participants were excluded because whether they had been referred to a medical facility was unknown

### Consequences of the referrals

Of the 72 people who were referred to a medical facility with or without a referral letter, 11 received replies from a medical doctor. In addition, medical information letters were sent from medical facilities to the NGO for two cases who were not recorded as “referred.” The date of the medical visit was known for five of these cases; only one person visited a medical facility within a month of the free consultation. Of the 13 cases, no urgent and/or serious disease was found; however, three people were diagnosed with valvular regurgitation, atrial fibrillation, or a thyroid tumor after further investigation, and were placed under medical observation.

## Discussion

We conducted secondary analyses of data for a sample of diverse foreign residents who attended free medical consultations offered by an NGO in various locations in Aichi Prefecture. Four fifths of the participants were covered by Japanese public insurance; however, in the consultations with the volunteer doctors over one fourth of the participants were advised to visit a medical facility. It was assumed that these individuals generally experienced barriers to accessing the formal health-care system. Risk groups regarding being advised to visit a medical facility or being referred to a medical facility were found to be people who are uninsured, people who are unemployed, and students. Of the 72 cases who were referred to a medical facility, the NGO received reply letters from medical facilities for just 11 cases.

Insurance coverage was an associated factor with being advised to visit a medical facility, which was our hypothetical indicator of access to health care. The finding is consistent with a previous study of Nepalese migrants, although indicators are not the same. In the Nepali study, 35% of the participants had not paid the insurance premium for a year or more [8]. This figure is higher than the proportion of no coverage of a public health insurance scheme in our current study (19%). Our team studied unmet health care needs among Filipino women, and the insurance coverage was not associated probably due to the high coverage (92%) among the target group [22]. Japan implemented universal public insurance coverage in 1961 [3], and in 2012 implemented a law to allow every person living in Japan, regardless of their nationality (but excepting visitors staying for three months or shorter), to receive coverage under a Japanese public insurance scheme [27]. As there are numerous insurance schemes in Japan, it is difficult to obtain exact figures regarding coverage among foreign residents. According to reports from some local governments and NGOs, however, it has been estimated that approximately 80% of foreign residents are covered by a Japanese public insurance scheme [7, 37]. This shows a dramatic improvement in coverage in recent decades, as in 2004 a survey conducted by a local government reported that this figure was 64.4% [38]. Our findings contribute to know that the low coverage rate among foreign residents in Japan, where there is a universal health insurance coverage policy, and to prove a social concern that no insurance coverage is a barrier to health care.

Previous studies on access to health care among Nepalese residents in Japan did not find associations between access variables and employment status [8]. In contrast, our study suggested that unemployed people represent a high-risk group. Although it is difficult to speculate the underlying reasons, income and social network, which was unknown in our dataset, may have an important effect. In addition, students were found to represent another possible risk group. In May 2019, 312,214 foreign students were living in Japan [39]. Among these, 53.2% were studying at professional training colleges, university preparatory courses, or Japanese language institutes [39]; institutions for which the School Health and Safety Act generally does not apply. According to a 2009 survey conducted by the Association for the Promotion of Japanese Language Education, 66.5% of the respondents from Japanese language schools had experienced illness or injury since arriving in Japan; of these, 2.3% had been hospitalized, 39.8% had visited medical facilities, and 2.7% had consulted their school’s medical personnel [25]. Further, among this group the most frequent answer to a question regarding the types of treatment used was “self-medication” (44.3%) [40]. Meanwhile, in a survey of students in Japanese language schools in Tokyo, 48.7% of the surveyed students reported that they did not think they had access to doctors/health workers [23]. Foreign students in Japanese language schools have become a target population in regard to measures to address tuberculosis [41], with foreign-born people accounting for a major part of new cases among young generations [42]. The government’s “300,000 International Students Plan,” has led to the arrival in Japan of a cohort of foreign students with diverse characteristics. A survey conducted by a governmental organization suggested that over three-quarters of privately-funded foreign students hold part-time jobs, among which almost 70% work over 15 h per week [43]; working such relatively long hours while also attempting to fulfill education responsibilities might explain the health-care-related vulnerability among this group. Systems to accept foreign students should be improved. Japanese society needs to recognize the vulnerability of this group.

Another important finding was that, after volunteer doctors sent attendees to health-care providers in the formal health care system, few replies were obtained. There are at least two possible explanations for this. First, the patients may not have visited a medical facility as advised; and second, the doctors who received the patients did not issue medical information letters to the NGO. Among the returned letters, we found that some patients had been placed under observation. If these referred patients had not visited a medical facility within an appropriate time, their diagnosis would have been delayed. Practically, it is difficult to track referred patient and to check whether they contacted the formal health-care system. Under the government plan for “Promotion of Multicultural Coexistence,” various NGOs, along with local governments, are expected to take a role in addressing livability issues for foreign residents [44]. To ensure access to health-care among foreign residents, better communication between NGOs and the formal health care system is required.

We applied the concept of “unmet health care needs” [35]. However, due to the nature of secondary data, information available in the dataset might not have been the most ideal. Globally standardized questions were not asked. After careful observation, we assumed that participants being advised to visit a medical facility and being referred to a medical facility indicated underuse of the formal health care system, and decided to apply this as negative indicators of access to health care in limited information. Both supply-side and demand-side characteristics are considered associated with access to health care in theories and frameworks of access to health care. We could not include supply-side variables in our model because such information was not available in our dataset. Although the availability of specific supports, for example, translators, might be an issue, considering the Japanese health care system and the density of health facilities, health-care providers’ physical availability in urban areas does not matter much. Therefore, even supply-side variables were unavailable, we believe that our analyses were valid.

Cases in the dataset were not randomly sampled from all foreign residents in Aichi Prefecture. Although the free consultations are open to everybody, people with similar attributes might have attended as a group. Consequently, the participant characteristics determined in this research may have been biased. However, compared with the proportions of countries of origin listed for residents of Aichi Prefecture in the government statistics for the corresponding years [45], our data can be considered to better reflect the target population in the prefecture. A venue-based approach for sampling hard-to-reach populations would be an acceptable method of obtaining more accurate data [46, 47]. It was reported that using only census and vital statistics may not be suitable for capturing the reality of the health situations among foreign residents [48]. Official data describing access to health care among foreign people remain limited; for example, to the best of our knowledge there are no official data regarding the prevalence of uninsured foreign residents. As previous studies have suggested [7, 23, 49], migrants are likely to be mobile, meaning it may be difficult to study foreign residents through mail surveys with random sampling. A limited number of studies have been published regarding access to health care among foreign residents in Japan. One recent study employed purposive sampling at restaurants to sample Nepalese participants [8]. Another study, which was not directly studied access to health care, sought to use random sampling, but only targeted Latin Americans in one city [7]. Thus, analysis of alternative data, such as that used in our study, is needed. Although the information we used was not collected for research purposes, our findings are nevertheless informative. As we have shown, analyzing data from NGOs can strengthen understanding of, and foster improvement in, access to health care and health outcomes. In the future, studies with randomly selected data, obtained through collaboration with local governments, will be required for more rigorous analyses.

There were some other limitations to this study. First, the data were entered by case (consultation), not by person. If the dataset includes many people who attended multiple consultations, the statistical findings may have been overestimated. However, because free consultations are held in different places in Aichi Prefecture over the course of the year, it is unlikely that the number of repeat visitors within  one year would be large. Second, obtaining reliable answers to some questions from some participants was difficult. For example, occupation was an open-ended, self-report answer, and volunteers (interviewers) sometimes needed to infer attendees’ jobs from their responses. As a result, the occupations of nearly 30% of the participants were unknown and were masked in the category of “No answer”, which may have influenced analyses.

Among possible risk groups we found regarding access to health care, those uninsured and the unemployed are considered hard-to-reach groups. It is considered that collaboration with NGOs, which is suggested by the government, should be essential to support these groups. Students were found another vulnerable group although they are insured by regulations. Japanese society needs to recognize the vulnerability of this group. Also, systems to accept international students should be improved. The current study was realized by analyzing NGO’s data, and such collaboration is necessary to generate evidence to support foreign residents in Japan.

## Conclusion

Secondary analysis of NGO’s activity records revealed the diversity of foreign residents living in Aichi Prefecture who require informal, free health consultations. Although the majority of the participants were covered by Japanese health insurance, almost 30% were advised to visit a medical facility, which implied that they had limited access to the formal health-care system before availing of the NGO’s consultations. Being advised to visit a medical facility was associated with insurance coverage. In addition to unemployed people, students were found to be a risk group. We found that not only assumed hard-to-reach groups, such as those uninsured and unemployed, but also those who are systematically accepted in the country, such as students, were considered likely to have a barrier to health care. Japanese society, including NGOs, should improve systems to accept them and also generate updated evidence about who is “left behind.”

## Data Availability

The datasets generated and/or analyzed during the current study are not publicly available due to agreement between the data owner (MICA) and the first author but are available from the first author on reasonable request.

## Abbreviations

CI: Confidence interval
MICA: Medical Information Center, Aichi
NGO: Non-governmental organization
NPO: Non-profit organization
PR: Prevalence ratio
UHC: Universal health coverage
